# Shape-persistent macrocycle with intraannular alkyl groups: some structural limits of discotic liquid crystals with an inverted structure

**DOI:** 10.1186/1860-5397-4-1

**Published:** 2008-01-09

**Authors:** Sigurd Höger, Jill Weber, Andreas Leppert, Volker Enkelmann

**Affiliations:** 1 Kekulé-Institut für Organische Chemie und Biochemie der Universität Bonn, Gerhard-Domagk-Str. 1, 53121 Bonn, Germany; 2 Institut für Technische Chemie und Polymerchemie, Universität Karlsruhe (TH), Engesserstr. 18, 76131 Karlsruhe, Germany; 3 Max-Planck-Institut für Polymerforschung, Ackermannweg 10, 55128 Mainz, Germany

## Abstract

The synthesis and thermal properties of new shape-persistent macrocycles of different sizes decorated with intraannular alkyl chains are described. The alkyl chain length is in all cases sufficient to cross the rings and to fill their interior completely. The investigation of the thermal behavior has shown that the smaller cycles do not exhibit thermotropic mesophases. Single crystal x-ray analysis indicates that the anisotropy in these compounds is too small to describe them as plates rather than spheres. For the larger macrocycles it is shown that longer adaptable substituents decrease the phase transition temperatures compared to previously described structures.

## Background

During the past several years, the interest in the design and study of shape-persistent macrocycles with an interior in the nanometer regime has considerably increased. From the structural point of view, most compounds are based on the phenylene, phenylene acetylene or phenylene butadiynylene backbone, or they contain a mixture of these structural elements. However, apart from meeting the synthetic challenge, the supramolecular chemistry of rigid rings is currently investigated with considerable effort. For example, shape-persistent macrocycles can act as host structures for appropriate guest molecules; they can form 1D aggregates (in solution or gas phase) or regular 2D lattices after deposition at defined surfaces [1–8]. Furthermore, shape-persistent macrocycles are interesting mesogens for discotic liquid crystalline materials [9–12].

The common design principle of conventional discotic liquid crystals (LCs) is a more or less rigid core (disk-like or macrocyclic) with peripheral flexible side groups that point outward [13–16]. They can be used for a variety of different optic and electronic applications, for example as materials for photovoltaics (in the columnar phase) [17–19] or as compensation layers in display technology (in the nematic phase) [20–22].

Recently, we could show that shape-persistent macrocycles with fixed intraannular side chains (e.g. **1**) can also exhibit liquid crystalline behavior [23–24]. Compound **1** is a shape-persistent macrocycle which is based on the phenylene-ethynylene-butadiynylene backbone. As it generally holds for rigid compounds, no matter if they are rod-like or cyclic, flexible side groups need to be added to keep these compounds tractable, i.e. soluble or/and meltable. For this purpose, **1** contains four long octadecyloxy side groups pointing inside the ring and eight propyloxy groups at the adaptable positions of the compound. The term adaptable means that the orientation of substituents at that position can be influenced by an external parameter [25–26].

**Figure 1:**
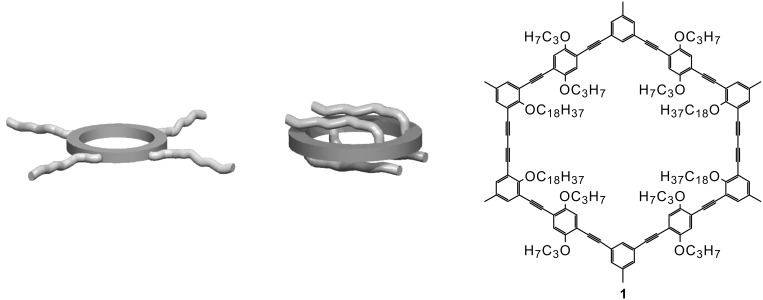
a) Design principle for common discotic liquid crystals; b) Design principle for discotic liquid crystals with an inverted structure; c) Structure of the shape-persistent macrocycle **1** containing extraannular methyl groups, intraannular octadecyloxy groups and adaptable propyloxy groups.

Compound** 1** melts at 134 °C to form a nematic mesophase that becomes isotropic at 159 °C. However, compared to all previously reported discotic LCs, this compound is composed of a *rigid periphery and the flexible side chains point inward*. Hence, **1** can be described as a discotic LC with an inverted structure (Figure 1) [23–24].

By comparing the structure of **1**, and other shape-persistent macrocycles, with their thermal behavior we could identify some preliminary guidelines for the observation of liquid crystallinity in those compounds. Among these is the necessity of the rings to fill their interior more or less with their own alkyl chains and the absence of bulky peripheral side groups, both in order to prevent an interlocking of the rings. However, the number of compounds that follow this new design principle is still rather limited. This was a motivation for us to synthesize additional macrocycles and to explore their thermal behavior.

Here we describe the synthesis and the thermal behavior of several new shape-persistent macrocycles with fixed intraannular alkyl groups. Their investigation is a further step towards obtaining a clearer structure-property relationship in these materials. Although the new compounds described in this paper differ in size and backbone structure, the presence of intraannular alkyl groups that fill the ring interior completely is common for all macrocycles, thus making them potential candidates for the observation of thermotropic liquid crystallinity according to the new design principle described above.

## Results and Discussion

### Synthesis

**Scheme 1:**
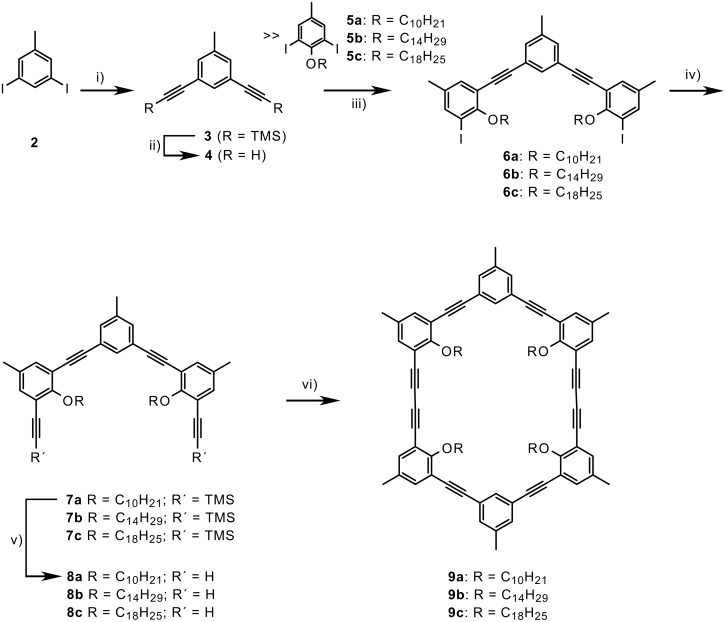
Synthesis of macrocycles with intraannular alkyl chains. i) TMS-acetylene, PdCl_2_(PPh_3_)_2_, CuI, NEt_3_/THF (97%); ii) K_2_CO_3_, MeOH/THF (87%); iii) PdCl_2_(PPh_3_)_2_, CuI, piperidine/THF (37-38%); iv) TMS-acetylene, PdCl_2_(PPh_3_)_2_, CuI, piperidine/THF (76-82%); v) K_2_CO_3_, MeOH/THF (89-97%); vi) CuCl, CuCl_2_, pyridine (12-16%).

Scheme 1 shows the synthesis of the macrocycles that have a reduced interior size compared to macrocycle **1**. 3,5-Diiodo-4-methylbenzene (**2**) was treated with trimethylsilyl(TMS)acetylene under standard Hagihara-Sonogashira coupling conditions and subsequently deprotected with K_2_CO_3_ in MeOH/THF. As expected, the Pd-catalyzed coupling reaction runs under milder conditions and with higher yields compared to the preparation of **3** from the corresponding dibromo compound [27]. Reaction of **4** with an excess of **5** [28], coupling of **6** with TMS-acetylene and deprotection, again with K_2_CO_3_ in MeOH/THF, gave the bisacetylenic half-ring **7**. The oxidative dimerization of **7** was performed by slow addition of a solution of **7** in pyridine to a suspension of CuCl and CuCl_2_ in the same solvent. Column chromatographic purification and repeated recrystallization from ethyl acetate gave the pure macrocycles not contaminated with higher oligomers, as determined by analytical gel permeation chromatography (GPC). The repeated purification process for all macrocycles was not optimized and is responsible for the rather low yield in the cyclization step.

**Scheme 2:**
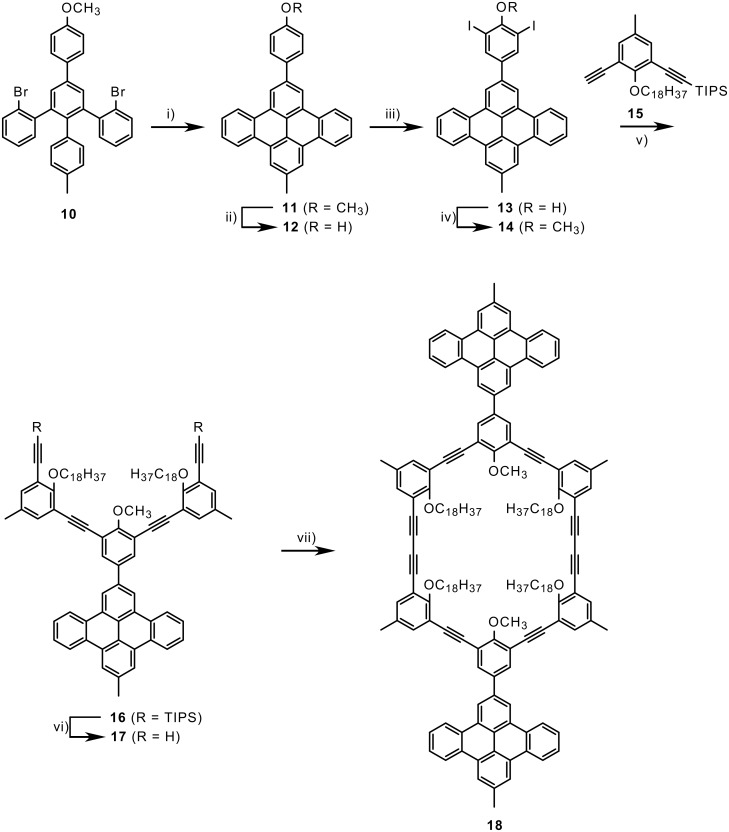
Synthesis of macrocycles with intraannular alkyl chains and extraannular PAH substituents. i) Pd(OAc)_2_, ligand, DBU, DMA (49%); BBr_3_, CH_2_Cl_2_ (99%); iii) I_2_/KI, ethylene diamine (quant.); iv) dimethyl sulfate, KOH, THF/water (50%); v) PdCl_2_(PPh_3_)_2_, CuI, piperidine/THF (71%); vi) Bu_4_NF, THF (97%); vii) CuCl, CuCl_2_, pyridine (28%).

Our recent progress in the synthesis of functionalized polycyclic aromatic hydrocarbons (PAHs) opened the question about the influence of the presence of PAH substituents on the phase behavior of these macrocycles [29–30]. A PAH has previously been incorporated into a shape-persistent macrocycle as a part of the rigid backbone [26]. The investigation of the thermal behavior of that compound led to the indication that PAHs could stabilize the thermotropic mesophases. However, reports about shape-persistent macrocycles with extraannular PAH substituents are absent. If these compounds exhibit liquid crystallinity, the question about the mesogenic element (ring or PAH or both) arises. Additionally, biaxial nematic phases might be observable [31–32]. The synthesis of the corresponding PAH building block and the subsequent macrocycle synthesis is displayed in Scheme 2.

Treating the aromatic dibromo compound **10** with Pd(OAc)_2_ (20 mol%) and Cy_2_P(2,2′MeO)biph (30 mol%) in DMA gave the corresponding dibenzonaphthacene in 40-45% yield. Although a detailed investigation of the Pd-catalyzed dehydrohalogenation is yet not completed, the yield is reproducible higher than with Pd(PPh_3_)_2_Cl_2_, as we reported before [29]. **11** was demethylated with BBr_3_, the resulting free phenol **12** iodinated with iodine and sodium iodide, and diiodide **13** realkylated with dimethylsulfate (model reactions with 4-methylanisole showed that a direct diiodination could not be obtained under the conditions we used; for example, with N-iodosuccinimide and FeCl_3_ we could obtain cleanly 2-iodo-4-methylanisole). Pd-catalyzed Hagihara-Sonogashira coupling with **15**, deprotection of the triisopropylsilyl (TIPS) groups and oxidative cyclodimerization under pseudo high-dilution conditions gave the macrocycle **18**.

**Scheme 3:**
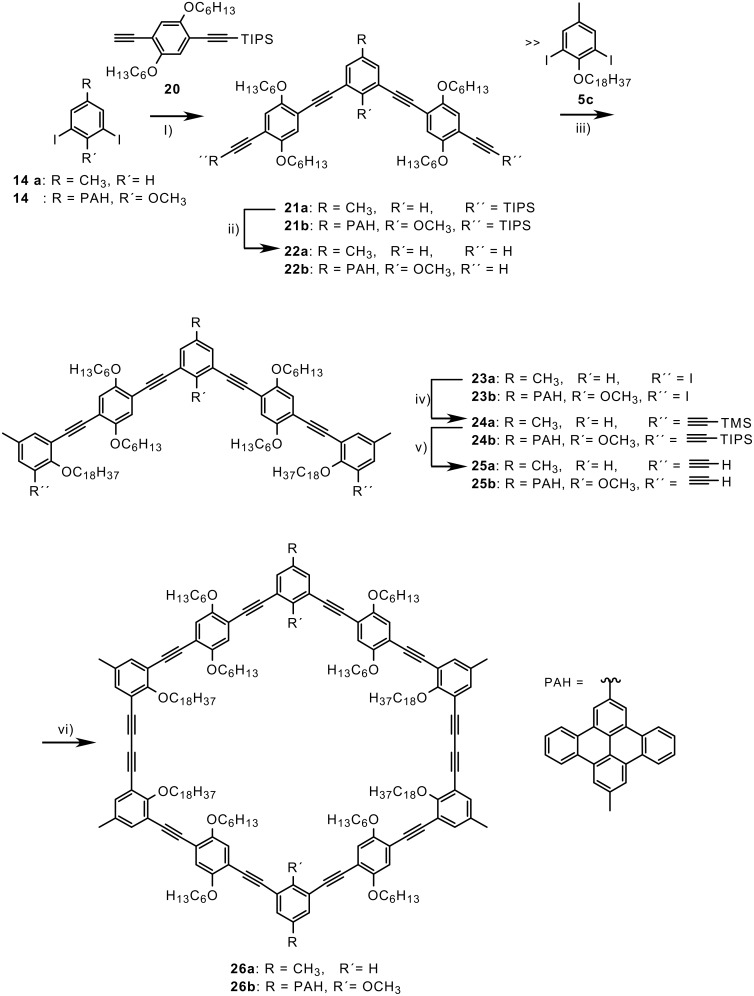
Synthesis of macrocycles with adaptable hexyloxy groups. i) PdCl_2_(PPh_3_)_2_, CuI, piperidine/THF (76-78%); ii) Bu_4_NF, THF (60-99%); iii) PdCl_2_(PPh_3_)_2_, CuI, piperidine/THF (46-48%); iv) TMS (TIPS)-acetylene, PdCl_2_(PPh_3_)_2_, CuI, piperidine/THF (64-98%); v) K_2_CO_3_, MeOH/THF or Bu_4_NF, THF (95-99%); vi) CuCl, CuCl_2_, pyridine (7-23%).

Based on the comparison of the thermal behavior of **9** and **18** with **1** we intended to prepare also isomers of the latter containing longer adaptable side groups, with and without extraannular PAH substituents (Scheme 3). Pd-catalyzed coupling of the diiodo compound **14** with the mono protected bisacetylene **20**, deprotection of the acetylenes with TBAF and subsequent coupling with an excess of the diiodo compound **5c** gave the diiodide **23**. Hagihara-Sonogashira coupling of **23** with an excess of TMS acetylene or TIPS acetylene, respectively, base(fluoride)-catalyzed removing of the silyl groups and cyclodimerization of the bisacetylenes **25**, again under pseudo high-dilution conditions, gave the macrocycles **26a** and **26b**, respectively.

### Thermal behavior and X-ray structure

**Figure 2:**
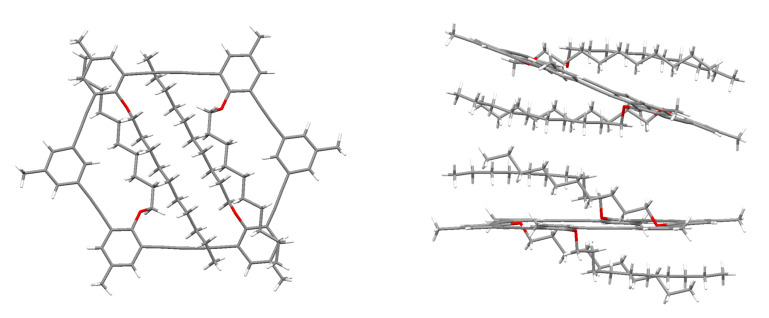
Single crystal X-ray structure of **9a** (solvent not shown): a) Top view; b) Side view of two molecules of **9a** in the crystal.

The thermal behavior of the macrocycles **9** and **18** was of special interest in order to evaluate if macrocycles with a reduced interior size (compared to **1**) also exhibit liquid crystallinity. All macrocycles **9** melt without decomposition (**9a**: 162 °C; **9b**: 132 °C; **9c**: 88 °C). As expected, with increasing alkyl chain length the melting points of the compound decreases and in the latter case it is even below 100 °C. However, the investigation of the three macrocycles **9** under the polarizing microscope showed that none of the compounds is thermotropic liquid crystalline. Under the assumption that the molecular structure in the solid state could help in understanding the absence of a mesophase, attempts were undertaken to perform a single crystal x-ray investigation on at least one of the compounds. By slow evaporation of a CH_2_Cl_2_ solution of **9a** single crystals suitable for x-ray analysis could be obtained. **9a** crystallizes as solvate with one molecule CH_2_Cl_2_ per macrocycle (not shown in Figure 2).

The asymmetric unit of **9a** contains two crystallographically independent (half)rings which are located on two centers of symmetry in the triclinic unit cell. These are not parallel to each other but inclined by an angle of about 25°. It should also be mentioned that the two macrocycles differ in the conformation of the alkyl chains. In one of them the side chains adopt an all-trans conformation whereas in the other two alkyl chains form a kink by a gauche conformation of the third bond (see Supporting Information File 1 for additional images). This is probably to fill the space between the rings effectively by enabling adjacent chains to be packed parallel to each other. The top view shows that the internal void of both rings is already filled with the first few carbon atoms of the intraannular alkyl chains (Figure 2, left). The remaining portions of the alkyl chains are located above and below the rigid phenyl acetylene backbone of the rings which is in both cases relatively flat. Although the crystal packing shows that the alkyl chains are sandwiched between the macrocycles, there exist still some close contacts between the π-systems of adjacent macrocycles (e.g. C65-C72: 3.419 Å, see Supporting Information File 1 for additional images). An interlocking of the molecules through entanglement between rigid and flexible molecule parts cannot be observed. In addition, the extraannular methyl groups at the ring corners are also not bulky enough to extend into the interior of neighbored rings. Therefore, a physical crosslinking of the molecules, as it was supposed to be responsible for the absence of a mesophase in other shape-persistent rings, can be excluded. However, different to the previously investigated compounds the lateral extension of the macrocycles **9** is rather small. The distance between the two diacetylene bridges in **9** is around 1.2 nm compared to 2.3 nm in **1**. Since the intraannular alkyl chains are located above and below the macrocyclic framework, the anisotropy of the compound is remarkably lower as in **1**, leading to an aspect ratio that does not support the mesophase formation. Interestingly, our expectation to observe LC behavior when extraannular PAH substituents were attached to this macrocycle was not fulfilled. Also for **18** no mesophase could be observed. Two reasons might account for that. First, the stronger interaction between the exocyclic parts of the rings leads to a significantly higher melting point (206 °C), above the stability range of a possible mesophase. And second, the ability of the PAH substituents to rotate freely might lead to an even more unfavorable aspect ratio of the molecule. In any case, although only a few compounds with intraannular alkyl groups have been investigated, their thermal behavior indicates that the tendency of smaller rings to exhibit mesomorphic behavior is less pronounced compared to the larger rings (as e.g. **1**). Investigations on derivatives of **9** with additional extraannular flexible side groups are ongoing and will be reported elsewhere.

Apart from the size of the macrocycles and the size of the extra- and intraannular substituents, the thermal behavior of the compounds can be influenced by the adaptable substituents. Previous investigations have shown that the presence or absence of these substituents have a dramatic effect on the mesophase formation [24]. For example, when the eight adaptable propyloxy groups of **1** are removed, the material has a higher melting point (185 °C) and does not exhibit a mesophase. Contrary, derivatives of **1** with longer adaptable side groups are yet not investigated. Two compounds of that structure are described in this work. Macrocycle **26a** melts isotropically at 98 °C (first heating, *ΔH* = 85.2 kJ/mol). However, cooling of the isotropic melt leads to the formation of a (monotropic) nematic phase at 90 °C (*ΔH* = 2.2 kJ/mol) that recrystallizes at 34 °C. Macrocycle **26b** has a considerable higher melting point of 187 °C (first heating, *ΔH* = 86.3 kJ/mol). Also in this case the melt is isotropic but becomes birefringent at 160 °C, as observed by polarizing microscopy. This latter transition could not be observed by DSC. A recrystallization of the material during the cooling process could also not be observed, either by polarizing microscopy or by DSC measurements (for the aggregation of **26b** in solution see Supporting Information File 1).

These data show that the adaptable substituents are another parameter to fine tune the thermal behavior of discotic liquid crystals with an inverted structure. This parameter is absent in conventional discotic liquid crystals. As expected, longer adaptable alkyl substituents (compared to **1**) decrease the melting point of the macrocycles but at the same time also narrow the temperature range of the mesophase so that for **26** only monotropic mesophases could be observed. It is worth noting that also the attachment of PAH substituents does not in this particular case lead to an observation of an enantiotropic mesophase. This behavior is opposite to a previously reported example where a PAH unit incorporated into the macrocyclic backbone strongly stabilizes the mesophase.

## Conclusion

In conclusion, we have prepared several new shape-persistent macrocycles of different sizes that contain intraannular alkyl chains of sufficient length to cross the whole rings and to fill their interior. The investigation of the thermal behavior has shown that the smaller cycles do not exhibit thermotropic mesophases. Although single crystal x-ray analysis has proven that these compounds fulfill in principle our previously described design principle for discotics with an inverted structure, the aspect ratio of the macrocycles with their alkyl surrounding is too small to describe them as plates rather than spheres.

For the larger macrocycles we could show that longer adaptable substituents decrease the phase transition temperatures. This parameter, absent in conventional discotics, is another tool to fine tune the thermal behavior of these materials. 

### Selected crystallographic data

Data collection with Mo Kα radiation on a Nonius KCCD diffractometer. Selected crystal data: 9a CH_2_Cl_2_ (T = 120 K): triclinic, P−1, a = 15.3188(6), b = 16.3517(6), c = 20.0689(7) Å, α = 88.9288(13), β = 69.1591(15), γ = 70.4677(14) °, V = 4399.0(3) Å^3^, Z = 2, D_x_ = 1.089 g cm^−3^, 54439 reflections measured, 15014 unique reflections (R_int_ = 0.072), 6315 reflections observed (I > 3σ(I)); the structure was solved by direct methods (Shelxs) and refined by full matrix least-squares analyses on F with anisotropic temperature factors for C, O, Cl. The H atoms were included with fixed isotropic temperature factors in the riding mode. R = 0.0390, R_w_ = 0.0464. CCDC-644365 contains the supplementary crystallographic data for this paper. These data can be obtained free of charge via http://www.ccdc.cam.ac.uk/conts/retrieving.html, or from the Cambridge Crystallographic Data Centre, 12 Union Road, Cambridge CB2 1EZ, UK; fax: (+44)1223-336033; or email: deposit@ccdc.cam.ac.uk.

## Supporting Information

File 1 Shape-persistent macrocycles with intraannular alkyl groups: synthesis and x-ray structure. The data provided describe the synthesis and characterization of the different macrocycles.
