# Supplementary material for: Shape-persistent macrocycle with intraannular alkyl groups: some structural limits of discotic liquid crystals with an inverted structure
Source: Beilstein J Org Chem. 2008 Jan 9;4:1. doi: 10.1186/1860-5397-4-1 (PMC2238743; doi:10.1186/1860-5397-4-1)
Supplement: File 1 — Shape-persistent macrocycles with intraannular alkyl groups: synthesis and x-ray structure. The data provided describe the synthesis and characterization of the different macrocycles. [file Beilstein_J_Org_Chem-04-01-s001.pdf]

Shape-persistent macrocycles with intraannular alkyl groups: synthesis and x-ray structure

Jill Weber[§2], Andreas Leppert[§2], Volker Enkelmann[3] and Sigurd Höger*[1]

Address: [1]Kekulé-Institut für Organische Chemie und Biochemie der Universität Bonn, Gerhard-Domagk-Str. 1, 53121 Bonn, Germany and [2]Institut für Technische Chemie und Polymerchemie, Universität Karlsruhe (TH), Engesserstr. 18, 76131 Karlsruhe, Germany and [3]Max-Planck-Institut für Polymerforschung, Ackermannweg 10, 55128 Mainz, Germany

Email: Sigurd Höger* - hoeger@uni-bonn.de; Jill Weber – weberjill@gmail.com; Andreas Leppert - andreas.leppert@ipoc.uni-stuttgart.de; Volker Enkelmann - ve@mpip-mainz.mpg.de

*Corresponding author.

[§]These authors contributed equally to this work

Supporting Information

Experimental part

General. Reactions requiring an inert gas atmosphere were conducted under argon, and the glassware was oven-dried (140 °C). THF was distilled from potassium prior to use. Triethylamine, piperidine and pyridine were distilled over CaH_2 and stored under argon. Commercially available chemicals were used as received. Thin-layer chromatography was performed on aluminum plates precoated with Merck 5735 silica gel 60 F_{254}. Column chromatography was performed with Merck silica gel 60 (230 - 400 mesh). Radial chromatography was performed on a chromatotron (Harrison research) using silica gel/gypsum including fluorescence indicator (Merck) as stationary phase. ^{1}H NMR and ^{13}C NMR spectra were recorded on a Bruker DPX 250 or AC 300 spectrometer (250 and 300 MHz for ^{1}H). Chemical shifts are given in ppm, referenced to residual proton resonances of the solvents. Gel permeation chromatograms were measured in THF (flow rate 1 mL min^{-1}) at room temperature, using a combination of two styragel columns (porosity 10^3, 10^5) and an UV detector operating at λ = 254 nm. The molecular weight was obtained from polystyrene calibrated SEC columns. The matrix-assisted laser desorption ionization time-of-flight (MALDI-TOF) mass spectroscopy measurements were carried out on a Bruker reflex spectrometer. FAB and EI mass spectra were recorded on a Finnigan MAT 90 machine. Melting points were measured with a Reichert hot stage apparatus and are uncorrected. DSC measurements were performed on a Mettler DSC 30, heating and cooling rates: 10 K min^{-1}.

3: $Pd(PPh_3)_2Cl_2$ (175 mg) and CuI (87 mg) were added to a solution of **2** [1] (3.00 g, 8.72 mmol) and in triethylamine/THF (5:1) (120 mL). TMS-acetylene (3.41 g, 8.55 mmol) was added and the solution was stirred for 18 h at room temperature and poured into ether and water. The organic phase was separated and extracted with water, sat. NH_4Cl solution, water, and brine. After drying over $MgSO_4$ and evaporation of the solvent the crude product was chromatographed over silica gel with petroleum ether as the eluent (R_f (petroleum ether) = 0.50) to give **3** as a slightly yellow solid (2.41 g, 97 %). ^{1}H-NMR (250 MHz, CD_2Cl_2): δ = 7.36-7.34 (m, 1 H), 7.24-7.22 (m, 2 H), 2.29 (s, 3 H), 0.24 (s, 18 H).

4: K_2CO_3 (3.40 g, 24.61 mmol) was added to a solution of **3** (2.33 g, 8.20 mmol) in MeOH/THF (1:1) (30 mL). The solution was stirred for 18 h at room temperature and

then poured into ether and water. The organic phase was separated and extracted with water, 10 % acetic acid, water, and brine. After drying over $MgSO_4$ and evaporation of the solvent the crude product was chromatographed over silica gel with petroleum ether as the eluent (R_f (petroleum ether) = 0.55) to give **3** as a slightly yellow oil (1.00 g, 87 %). ^{1}H-NMR (250 MHz, CD_2Cl_2): δ = 7.42-7.40 (m, 1 H), 7.32-7.30 (m, 2 H), 3.12 (s, 2 H), 2.32 (s, 3 H).

6: General procedure: $Pd(PPh_3)_2Cl_2$ (15 mg) and CuI (8 mg) were added to a solution of **4**, **5** and PPh_3 (15 mg) in THF/piperidine (1:2; 15 ml) and the mixture was stirred for 18 h at 60 °C. After cooling to room temperature the mixture was poured into ether and water. The organic layer was extracted with water and brine and dried over $MgSO_4$. The organic phase was separated and extracted with water, 10 % acetic acid, water, 10 % aqueous sodium hydroxide, water, and brine. After drying over $MgSO_4$ and evaporating the solvent excess of **5** was removed by column chromatography (petroleum ether/CH_2Cl_2 (gradient 10:1 to 2:1)) and the product was purified by radial chromatography over silica gel/gypsum. **6a**: **4** (100 mg, 0.71 mmol) and **5a** (3.48 g, 6.96 mmol) yielded **6a** (242 mg, 38 %) as a slightly yellow oil (petroleum ether/CH_2Cl_2 (2:1), R_f = 0.63).^{1}H-NMR (250 MHz, CD_2Cl_2): δ = 7.63-7.60 (m, 2 H), 7.49-7.47 (m, 1 H), 7.35-7.33 (m, 2 H), 7.31-7.28 (m, 2 H), 4.18 (t, J = 6.5 Hz, 4 H), 2.37 (s, 3 H), 2.28 (s, 6 H), 1.95 – 1.82 (m, 4 H), 1.62-1.48 (m, 4 H), 1.42 – 1.12 (m, 24 H), 0.81 (t, J = 6.7 Hz, 6 H). MS (FAB): 884.1 (45 %) [M+]. **6b**: **4** (104 mg, 0.74 mmol) and **5b** (3.97 g, 7.13 mmol) yielded **6b** (280 mg, 37 %) as a white solid (petroleum ether/CH_2Cl_2 (4:1), R_f = 0.62).^{1}H-NMR (250 MHz, CD_2Cl_2): δ = 7.63-7.60 (m, 2 H), 7.49-7.47 (m, 1 H), 7.35-7.33 (m, 2 H), 7.31-7.28 (m, 2 H), 4.18 (t, J = 6.5 Hz, 4 H), 2.37 (s, 3 H), 2.28 (s, 6 H), 1.95 – 1.82 (m, 4 H), 1.62-1.48 (m, 4 H), 1.42 – 1.12 (m, 40 H), 0.81 (t, J = 6.7 Hz, 6 H). MS (FAB): 996.2 (21 %) [M+]. **6c**: **4** (103 mg, 0.74 mmol) and **5c** (4.37 g, 7.13 mmol) yielded **6c** (314 mg, 38 %) as a white solid (petroleum ether/CH_2Cl_2 (4:1), R_f = 0.70).^{1}H-NMR (250 MHz, CD_2Cl_2): δ = 7.63-7.60 (m, 2 H), 7.49-7.47 (m, 1 H), 7.35-7.33 (m, 2 H), 7.31-7.28 (m, 2 H), 4.18 (t, J = 6.5 Hz, 4 H), 2.37 (s, 3 H), 2.28 (s, 6 H), 1.95 – 1.82 (m, 4 H), 1.62-1.48 (m, 4 H), 1.42 – 1.12 (m, 56 H), 0.81 (t, J = 6.7 Hz, 6 H). MS (FAB): 1108.3 (44 %) [M+].

7: General procedure. $Pd(PPh_3)_2Cl_2$ (6 mg) and CuI (3 mg) were added to a solution of **6** and PPh_3 (6 mg) in THF/piperidine (1:1; 18 ml) and the mixture was heated to 60

°C. TMS-acetylene (250 mg; 2.50 mmol) was added and the mixture was stirred for 18 h. After cooling to room temperature the mixture was poured into ether and water. The organic layer was extracted with water and brine and dried over $MgSO_4$. After evaporation of the solvent, **7** was purified by radial chromatography over silica gel/gypsum. 7**a**: **6a** (200 mg, 0.23 mmol) yielded **7a** (152 mg, 81 %) as a slightly yellow oil (petroleum ether/CH_2Cl_2 (10:1), R_f = 0.25).^{1}H-NMR (250 MHz, CD_2Cl_2): δ = 7.50-7.47 (m, 1 H), 7.35-7.32 (m, 2 H), 7.29-7.25 (m, 2 H), 7.24-7.21 (m, 2 H), 4.21 (t, *J* = 6.4 Hz, 4 H), 2.37 (s, 3 H), 2.27 (s, 6 H), 1.90 – 1.77 (m, 4 H), 1.62 – 1.48 (m, 4 H), 1.42-1.15 (m, 24 H), 0.86 (t, *J* = 6.7 Hz, 6 H), 0.27 (s, 18 H). MS (FAB): 824.4 (23 %) [M+]. 7**b**: **6b** (250 mg, 0.25 mmol) yielded **7b** (181 mg, 76 %) as a slightly yellow solid (petroleum ether/CH_2Cl_2 (4:1), R_f = 0.38).^{1}H-NMR (250 MHz, CD_2Cl_2): δ = 7.50-7.47 (m, 1 H), 7.35-7.32 (m, 2 H), 7.29-7.25 (m, 2 H), 7.24-7.21 (m, 2 H), 4.21 (t, *J* = 6.4 Hz, 4 H), 2.37 (s, 3 H), 2.27 (s, 6 H), 1.90 – 1.77 (m, 4 H), 1.62 – 1.48 (m, 4 H), 1.42-1.15 (m, 40 H), 0.86 (t, *J* = 6.7 Hz, 6 H), 0.27 (s, 18 H). 7**c**: **6c** (294 mg, 0.27 mmol) yielded **7a** (230 mg, 82 %) as a slightly yellow solid (petroleum ether/CH_2Cl_2 (4:1), R_f = 0.43).^{1}H-NMR (250 MHz, CD_2Cl_2): δ = 7.50-7.47 (m, 1 H), 7.35-7.32 (m, 2 H), 7.29-7.25 (m, 2 H), 7.24-7.21 (m, 2 H), 4.21 (t, *J* = 6.4 Hz, 4 H), 2.37 (s, 3 H), 2.27 (s, 6 H), 1.90 – 1.77 (m, 4 H), 1.62 – 1.48 (m, 4 H), 1.42-1.15 (m, 56 H), 0.86 (t, *J* = 6.7 Hz, 6 H), 0.27 (s, 18 H). MS (FAB): 1048.8 (95 %) [M+].

8: General procedure. 7 was dissolved in THF (6-8 ml) and K_2CO_3 (0.10 g,) and MeOH (8-10 ml) were added. The mixture was stirred for 18 h at room temperature, poured into ether and water and the organic phase was washed with water and brine. After drying over $MgSO_4$ and evaporating the solvent, **8** was purified by column chromatography. **8a**: **7a** (134 mg, 0.16 mmol) yielded **8a** (106 mg, 95 %) as a slightly yellow solid (petroleum ether/CH_2Cl_2 (2:1), R_f = 0.29).^{1}H-NMR (250 MHz, CD_2Cl_2): δ = 7.50-7.47 (m, 1 H), 7.35-7.33 (m, 2 H), 7.33-7.30 (m, 2 H), 7.27-7.25 (m, 2 H), 4.17 (t, *J* = 6.5 Hz, 4 H), 3.30 (s, 2 H), 2.37 (s, 3 H), 2.28 (s, 6 H), 1.90 – 1.77 (m, 4 H), 1.60 - 1.46 (m, 4 H), 1.40 - 1.08 (m, 24 H), 0.88 (t, *J* = 6.6 Hz, 6 H). **8b**: **7b** (168 mg, 0.18 mmol) yielded **8b** (137 mg, 97 %) as a slightly yellow solid (petroleum ether/CH_2Cl_2 (4:1), R_f = 0.39).^{1}H-NMR (250 MHz, CD_2Cl_2): δ = 7.50-7.47 (m, 1 H), 7.35-7.33 (m, 2 H), 7.33-7.30 (m, 2 H), 7.27-7.25 (m, 2 H), 4.17 (t, *J* = 6.5 Hz, 4 H), 3.30 (s, 2 H), 2.37 (s, 3 H), 2.28 (s, 6 H), 1.90 – 1.77 (m, 4 H), 1.60 - 1.46 (m, 4 H), 1.40 - 1.08 (m, 40 H), 0.88 (t, *J* = 6.6 Hz, 6 H). MS (FAB): 792.6 (31 %) [M+]. **8c**: **7c**

(213 mg, 0.20 mmol) yielded **8c** (165 mg, 89 %) as a white solid (petroleum ether/CH_2Cl_2 (4:1), R_f = 0.24).^{1}H-NMR (250 MHz, CD_2Cl_2): δ = 7.50-7.47 (m, 1 H), 7.35-7.33 (m, 2 H), 7.33-7.30 (m, 2 H), 7.27-7.25 (m, 2 H), 4.17 (t, J = 6.5 Hz, 4 H), 3.30 (s, 2 H), 2.37 (s, 3 H), 2.28 (s, 6 H), 1.90 – 1.77 (m, 4 H), 1.60 - 1.46 (m, 4 H), 1.40 - 1.08 (m, 56 H), 0.88 (t, J = 6.6 Hz, 6 H). MS (FAB): 904.6 (51 %) [M+].

9: General procedure. A solution of **8** in pyridine (9 ml) was added to a slurry of CuCl/$CuCl_2$ in pyridine within 96 h at room temperature and stirred for additional 16 h. The reaction mixture was poured into CH_2Cl_2/water and the organic phase was extracted with water, NH_3 solution (25 %), water, HOAc (10 %), water, NaOH solution (10 %), water and brine. After drying over $MgSO_4$ and evaporating the solvent, **9** was purified by column chromatography and subsequent recrystallization from ethyl acetate. **9a**: **8a** (95 mg, 0.14 mmol) and CuCl/$CuCl_2$ (0.78 g/0.15 g) in pyridine (19 ml) yielded **9a** (14 mg, 15 %) as colorless crystals (petroleum ether/CH_2Cl_2 (2:1), R_f = 0.16). m.p. 162 °C. ^{1}H-NMR (250 MHz, CD_2Cl_2): δ = 7.57 - 7.49 (m, 2 H), 7.35 – 7.31 (m, 8 H), 7.28 – 7.25 (m, 4 H), 4.32 (t, J = 6.4 Hz, 4 H), 2.38 (s, 6 H), 2.30 (s, 12 H), 1.96 – 1.84 (m, 8 H), 1.66 – 1.50 (m, 8 H), 1.44 – 1.00 (m, 48 H), 0.74 (t, J = 6.8 Hz, 12 H). MS (FAB): 1356.6 (87 %) [M+].

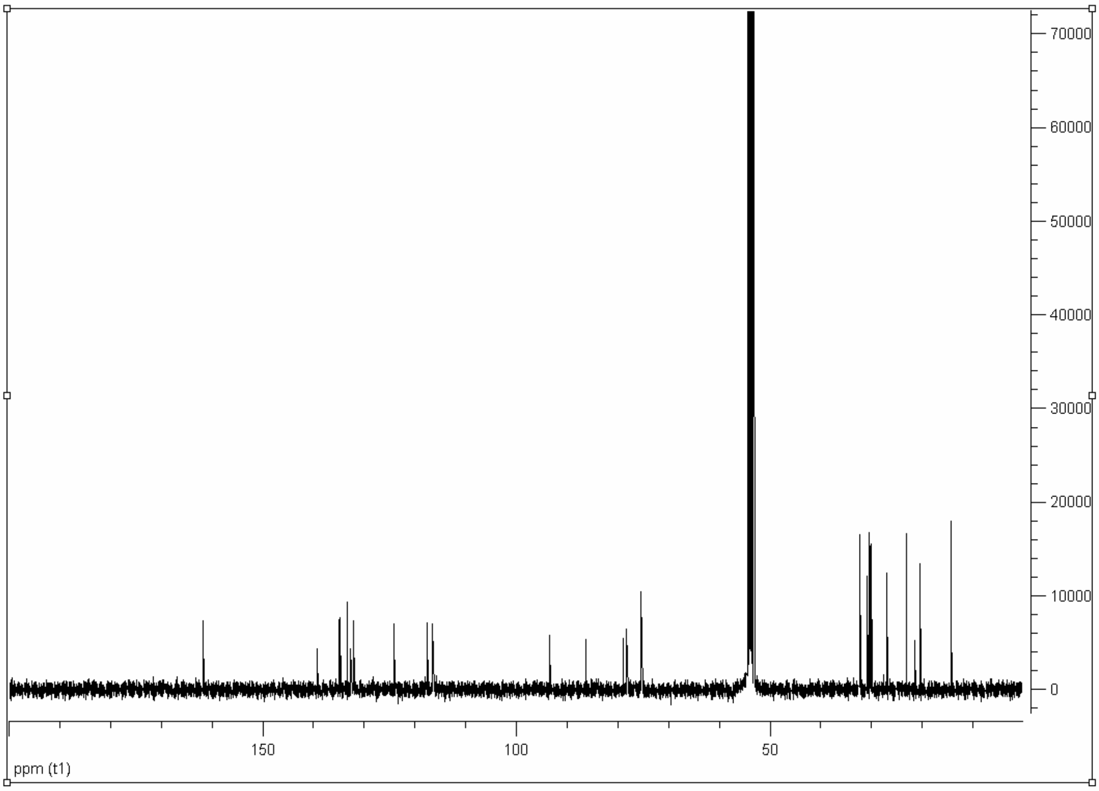

13C NMR spectrum of **9a.**

9b: **8b** (148 mg, 0.19 mmol) and CuCl/$CuCl_2$ (1.02 g/0.20 g) in pyridine (30 ml) yielded **9b** (18 mg, 12 %) as a colorless crystals (petroleum ether/CH_2Cl_2 (2:1), R_f = 0.17). m.p. 132 °C. MS (FAB): 1582.6 (28 %) [M+]. ^{1}H-NMR (250 MHz, CD_2Cl_2): δ = 7.57 - 7.49 (m, 2 H), 7.35 – 7.31 (m, 8 H), 7.28 – 7.25 (m, 4 H), 4.32 (t, J = 6.4 Hz, 4 H), 2.38 (s, 6 H), 2.30 (s, 12 H), 1.96 – 1.84 (m, 8 H), 1.66 – 1.50 (m, 8 H), 1.44 – 1.00 (m, 80 H), 0.85 (t, J = 6.8 Hz, 12 H).

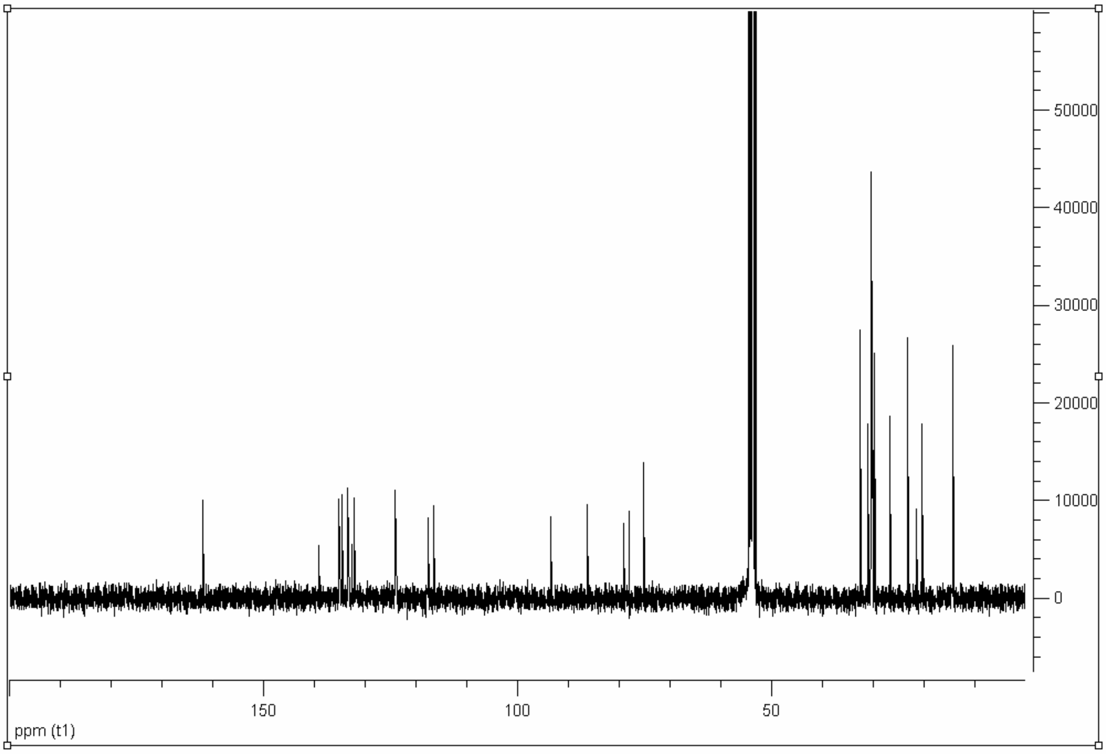

13C NMR spectrum of **9b.**

9c: **8c** (150 mg, 0.17 mmol) and CuCl/$CuCl_2$ (0.91 g/0.18 g) in pyridine (25 ml) yielded **9c** (23 mg, 16 %) as a colorless crystals (petroleum ether/CH_2Cl_2 (4:1), R_f = 0.16). m.p. 88 °C. ^{1}H-NMR (250 MHz, CD_2Cl_2): δ = 7.57 - 7.49 (m, 2 H), 7.35 – 7.31 (m, 8 H), 7.28 – 7.25 (m, 4 H), 4.32 (t, J = 6.4 Hz, 4 H), 2.38 (s, 6 H), 2.30 (s, 12 H), 1.96 – 1.84 (m, 8 H), 1.66 – 1.50 (m, 8 H), 1.44 – 1.00 (m, 112 H), 0.87 (t, J = 6.6 Hz, 12 H). MS (FAB): 1806.9 (10 %) [M+].

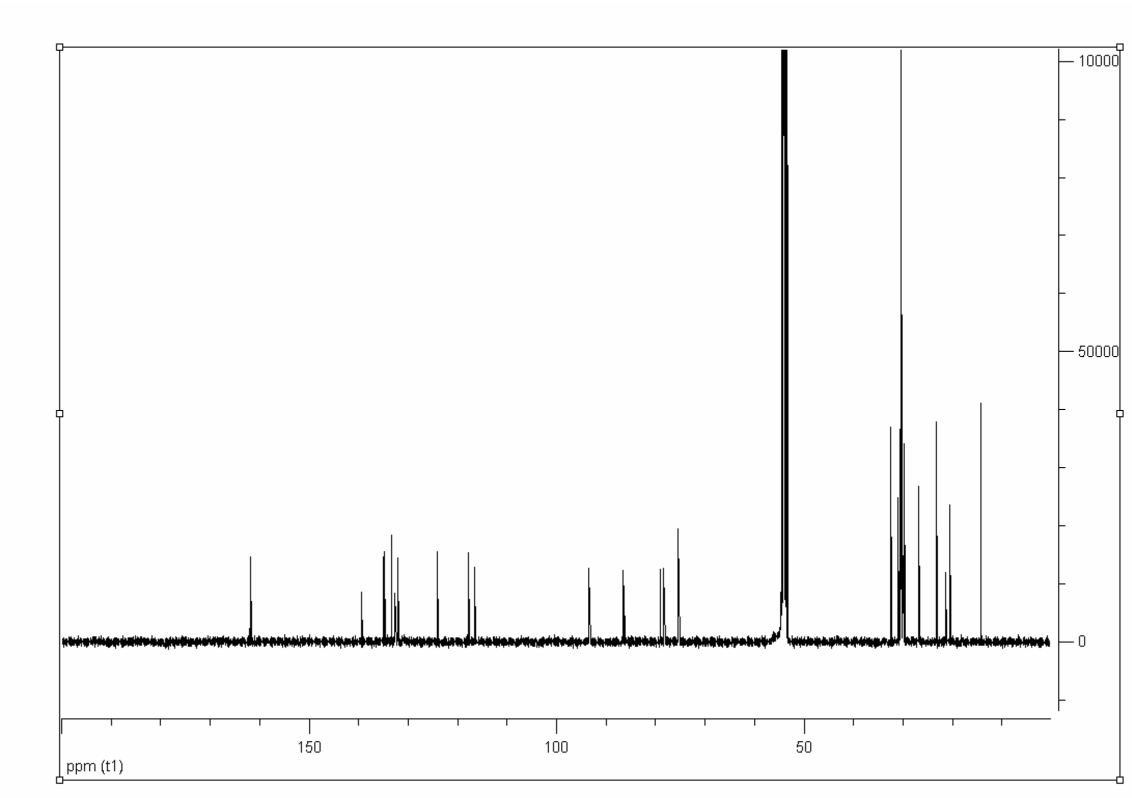

13C NMR spectrum of **9c.**

11: To a suspension of **10** (1.00 g, 1.71 mmol)[2], $Pd(OAc)_2$ (75 mg; 0.33 mmol) and dicyclohexyl(2',6'-dimethoxybiphenyl-2-yl)phosphine (215 mg; 0,51 mmol) in dimethylacetamid (DMA, 12 ml) was 1,8-diazabicyclo[5.4.0]undec-7-ene (DBU, 1.8 ml) added and the yellow mixture stirred at 160°C for 20 h. After cooling to room temperature, the black suspension was poured into $CHCl_3$ (100 ml) and washed with water and brine. After drying over $MgSO_4$ and evaporation of the solvent, **11** was purified by column chromatography (petroleum ether/CH_2Cl_2 (2:1), R_f = 0,63) and subsequent recrystallization from $CHCl_3$ to give 356 mg (49 %) of **11** as a colourless solid. (mp = 237 - 238°C). ^{1}H-NMR (250 MHz, CD_2Cl_2): δ = 9.08 (s, 2 H), 8.95 - 8.80 (m, 4 H), 8.76 (s, 2 H), 7.97 - 7.92 (m, 2 H), 7.80 – 7.74 (m, 4 H), 7.19 - 7.14 (m, 2 H), 3.94 (s, 3 H), 2.88 (s, 3 H).

12: To a suspension of **11** (0.75 g, 1.78 mmol) in CH_2Cl_2 (30 ml) was added dropwise a 1M solution of BBr_3 in CH_2Cl_2 (4.0 ml, 4.0 mmol) at -78 °C. After 1 h the mixture was warmed to room temperature and stirred for additional 20 h. The mixture was quenched by the addition of water (5 ml) and after stirring for 1 h the in CH_2Cl_2 was evaporated. The residue was treated with H_2O/MeOH (1:1, 30 ml) heated to reflux,

and cooled and stored for 20 h at 4 °C. The colorless precipitate was filtered off, washed with water and MeOH and dried in vacuum to yield 725 mg (99 %) of **12** as a colorless solid (CH_2Cl_2, R_f = 0,4). The compound was used for the next step without further purification. ^{1}H-NMR (250 MHz, THF-d8): δ = 8.92 (s, 2 H), 9.05 - 8.86 (m, 4 H), 8.84 (s, 1 H), 8.47 (s, 2 H), 7.94 – 7.86 (m, 2 H), 7.76 – 7.66 (m, 4 H), 7.03 – 6.95 (m, 2 H), 2.87 (s, 3 H).

13: To a suspension of **12** (0.82 g, 2.00 mmol) in THF/water/ethylene diamine (30 ml/0.6 ml/0.6 ml)) was added dropwise a solution of I_2/KI (1.15 g (4.42 mmol)/1.00 g (6.03 mmol)) in water (5.0 ml) at 40 °C. After additional stirring of the mixture for 15 h at 40 °C, the mixture was cooled to room temperature and 1M H_2SO_4 (10 ml) and $Na_2S_2O_3$ (0.81 g) were added. The mixture was evaporated to dryness and extracted with CH_2Cl_2 in a Soxhlet apparatus for 70 h. After evaporation of the solvent the yellow residue (1.41 g) was used without further purification (petroleum ether/CH_2Cl_2 (1:1), R_f = 0,67). ^{1}H-NMR (250 MHz, $C_2D_2Cl_4$): δ = 8.88 (s, 2 H), 8.87 – 8.75 (m, 4 H), 8.71 (s, 2 H), 8.23 (s, 2 H), 7.82 – 7.74 (m, 4 H), 5.93 (s, 1 H), 2.88 (s, 3 H).

14: To a suspension of **13** (1.35 g, crude product) and dimethyl sulfate (0.56 g, 4.43 mmol) in THF (20 ml) was added a solution of KOH in water (50 wt %, 1.2 g) and the mixture stirred for 20 h at 75 °C. After cooling to room temperature the mixture was poured into warm toluene and water. The organic layer was extracted with brine and dried over $MgSO_4$ and filtered hot. The solvent was evaporated and the residue refluxed in *i*-PrOH (10 ml), cooled to 4 °C, filtered off and dried in vacuum to give 694 mg (50 %) of **14** as a crème colored solid that was used without further purification (petroleum ether/CH_2Cl_2 (1:1), R_f = 0,88). ^{1}H-NMR (250 MHz, $C_2D_2Cl_4$): δ = 8.81 (s, 2 H), 8.80 – 8.74 (m, 4 H), 8.64 (s, 2 H), 8.28 (s, 2 H), 7.79 – 7.71 (m, 4 H), 4.00 (s, 3 H), 2.84 (s, 3 H). MS (EI): 673.9 [M+].

15: a) TIPS-acetylene (0.54 g, 2.88 mmol) were added to a solution of $Pd(PPh_3)_2Cl_2$ (59 mg), CuI (32 mg), **5c** (1.67 g, 2.73 mmol) and PPh_3 (60 mg) in THF/piperidine (2:1; 15 ml) and the mixture was stirred at room temperature for 18 h. CPDMS-acetylene (450 mg; 3.00 mmol) was added, the mixture was stirred for additional 20 h and then poured into ether and water. The organic phase was separated and extracted with water, 10 % acetic acid, water and brine. After drying over $MgSO_4$ and

evaporation of the solvent, the crude product was purified by column chromatography (petroleum ether/CH_2Cl_2 (gradient 4:1 to 2:1)) to give 668 mg (36 %) of CPDMS-protected **15** as a slightly yellow oil (petroleum ether/CH_2Cl_2 (2:1), R_f = 0,45). ^{1}H-NMR (250 MHz, CD_2Cl_2): δ = 7.25 - 7.19 (m, 2 H), 4.15 (t, 2 H, J = 6.9 Hz), 2.43 (t, 2 H, J = 7.0 Hz), 2.24 (s, 3 H), 1.90-1.73 (m, 4 H), 1.50-1.10 (m, 30 H), 1.15 (s, 21 H), 0,93 – 0.80 (m, 5 H, J = 6.6 Hz), 0.27 (s, 6 H).

b) K_2CO_3 (250 mg) was added to a solution of CPDMS-protected **15** (612 mg, 0.89 mmol) in THF/MeOH (1/1, 10 ml). After stirring for 2 h at room temperature, the mixture was poured into ether and water. The organic layer was extracted with water and brine and dried over $MgSO_4$. After evaporation of the solvent, **15** was purified by radial chromatography over silica gel/gypsum to give 306 mg (61 %) of **15** as a slightly yellow oil (petroleum ether/CH_2Cl_2 (10:1), R_f = 0,60). ^{1}H-NMR (250 MHz, CD_2Cl_2): δ = 7.27 – 7.21 (m, 2 H), 4.17 (t, 2 H, J = 6.8 Hz), 3.26 (s, 1 H), 2.25 (s, 3 H), 1.86-1.72 (m, 2 H), 1.55-1.15 (m, 30 H), 1.15 (s, 21 H), 0.89 (t, 3 H, J = 6.6 Hz).

16: $Pd(PPh_3)_2Cl_2$ (11 mg) and CuI (7 mg) were added to a solution of **14** (116 mg, 0.15 mmol), **15** (208 mg, 0.36 mmol) and PPh_3 (11 mg) in THF/piperidine (2:1; 9 ml) After stirring for 20 h at room temperature, the mixture was poured into ether and water. The organic layer was extracted with water, HOAc (10 %), water, NaOH solution (10 %), water and brine and dried over $MgSO_4$. After evaporation of the solvent, **16** was purified by radial chromatography over silica gel/gypsum (petroleum ether/CH_2Cl_2 (gradient 10:1 to 4:1)) to give 190 mg (71 %) of **16** as a colorless oil (petroleum ether/CH_2Cl_2 (4:1), R_f = 0,38). ^{1}H-NMR (250 MHz, CD_2Cl_2): δ = 8.91 – 8.90 (m, 2 H), 8.80-8.64 (m, 4 H), 8.56 (s, 2 H), 8.08 – 8.06 (m, 2 H), 7.75-7.65 (m, 4 H), 7.45 – 7.42 (m, 2 H), 7.35 – 7.32 (m, 2 H), 4.37 (t, J = 6.7 Hz, 4 H), 4.33 (s, 3 H), 2.80 (s, 3 H), 2.36 (s, 6 H), 2.02 - 1.86 (m, 4 H), 1.60 - 1.44 (m, 4 H), 1.35 – 0.93 (m, 64 H, Alkyl), 1.12 (s, 42 H), 0.99 (t, J = 6.7 Hz, 6 H).

17: Bu_4NF (1 M in THF, 0.75 ml) was added to a solution of **16** (190 mg, 0.12 mmol) in THF (1 ml). After stirring for 2 h at room temperature, the mixture was poured into CH_2Cl_2 and water. The organic layer was extracted with water and brine and dried over $MgSO_4$. After evaporation of the solvent, methanol was added to the residual oil and the mixture stored over night at 4 °C. The methanol was decanted and the procedure repeated in order to remove the silanol. The residue was dried in vacuum

to give 146 mg (97 %) of **17** as an slightly amber solid that was used as received (petroleum ether/CH_2Cl_2 (2:1), R_f = 0.41). ^{1}H-NMR (250 MHz, CD_2Cl_2): δ = 9.09 (s, 2 H), 8.94-8.81 (m, 4 H), 8.75 (s, 2 H), 8.10 (s, 2 H), 7.81 - 7.74 (m, 4 H), 7.45 – 7.42 (m, 2 H), 7.33- 7.30 (m, 2 H), 4.32 (t, *J* = 6.5 Hz, 4 H), 4.29 (s, 3 H), 3.33 (s, 2 H), 2.88 (s, 3 H), 2.33 (s, 6 H), 1.96 - 1.84 (m, 4 H), 1.62 - 1.46 (m, 4 H), 1.32 –0.92 (m, 56 H), 0.88 (t, *J* = 6.7 Hz, 6 H).

18: According to the general procedure for the synthesis of **9**: **17** (146 mg, 0.12 mmol) in pyridine (20 ml) was added to CuCl/$CuCl_2$ (0.67 g/0.09 g) in pyridine (60 ml). After the purification procedure described for **9** and an additional recrystallization from CH_2Cl_2 **18** (40 mg, 28 %) was obtained as a colorless crystals (petroleum ether/CH_2Cl_2 (1:1), R_f = 0.95). m.p. 206 °C. ^{1}H-NMR (250 MHz, CD_2Cl_2): δ = 9.00 (s, 4 H), 8.90 - 8.76 (m, 8 H), 8.68 (s, 4 H), 8.09 (s, 4 H), 7.80 - 7.72 (m, 8 H), 7.49 – 7.46 (m, 4 H), 7.34 – 7.31 (m, 4 H), 4.52 (t, *J* = 6.6 Hz, 8 H), 4.38 (s, 6 H), 2.86 (s, 6 H), 2.36 (s, 12 H), 2.08-1.94 (m, 8 H), 1.72 – 1.58 (m, 8 H), 1.56-1.00 (m, 112 H), 0.80 (t, *J* = 6.7 Hz, 6 H). MS (FAB): 2467.0 [100 %, M+].

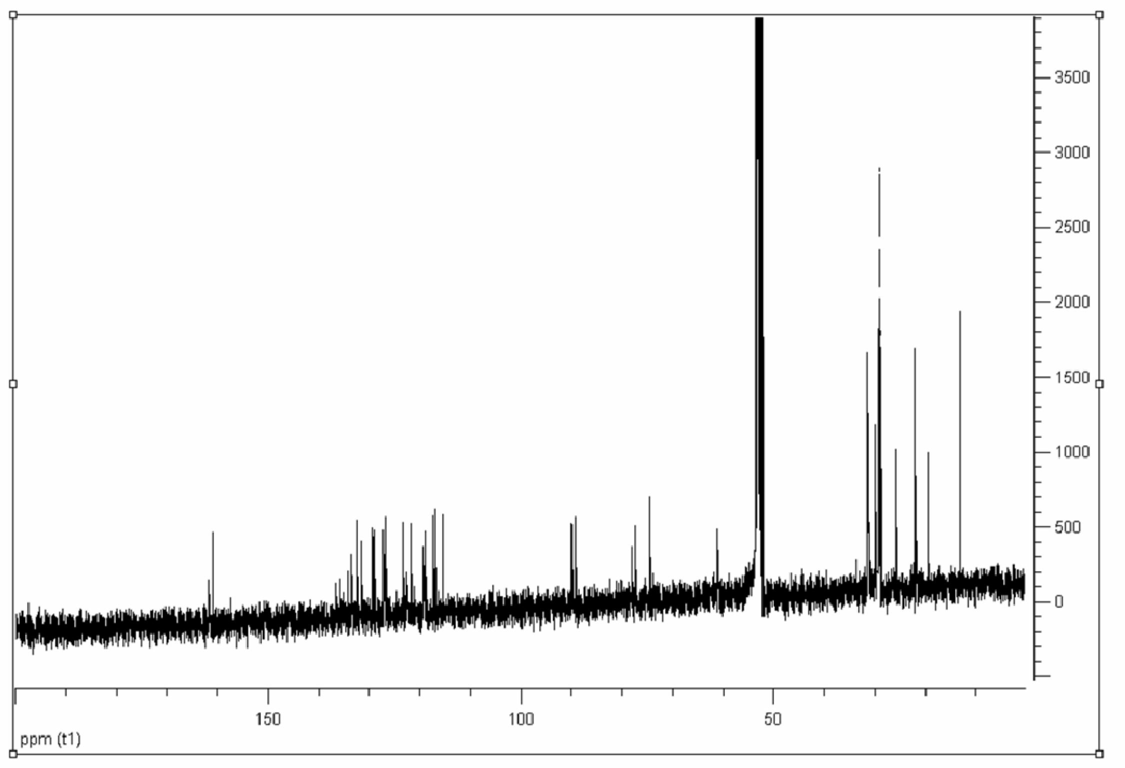

13C NMR spectrum of **18.**

21a: According to the synthesis of **16** from **20** (8.87 g; 18.37 mmol), 3,5-diiodotoluene (**14a**) (2.92 g; 8.5 mmol), piperidine (80 ml), $Pd(PPh_3)_2Cl_2$ (290 mg), CuI (150 mg) and PPh_3 (290 mg) **21a** was obtained as a slightly yellow solid (10.69 g, 78 %) (petroleum ether/CH_2Cl_2 (3:1), R_f = 0.48).^{1}H-NMR (250 MHz, $CDCl_3$): δ = 7.49 – 7.48 (m, 1 H), 7.32 – 7.31 (m, 2 H), 6.97 (s, 2 H), 6.96 (s, 2 H), 4.01 (t, J = 6.4 Hz, 4 H), 3.97 (t, J = 6.3 Hz, 4 H), 2.36 (s, 3 H), 1.90 - 1.70 (m, 8 H), 1.65 - 1.45 (m, 8 H), 1.45 - 1.20 (m, 16 H), 1.15 (s, 42 H), 0.90 (t, J = 6.7 Hz, 6 H), 0.88 (t, J = 6.7 Hz, 6 H).

21b: According to the synthesis of **16** from **20** (0.41 g; 0.85 mmol), **14** (0.27 g; 0.40 mmol), THF/piperidine (2/1; 15 ml), $Pd(PPh_3)_2Cl_2$ (19 mg mg), CuI (10 mg) and PPh_3 (19 mg) **21b** was obtained as a slightly yellow solid (0.42 g, 76 %) (petroleum ether/CH_2Cl_2 (4:1), R_f = 0.15).^{1}H-NMR (250 MHz, CD_2Cl_2): δ = 9.06 (s, 2 H), 8.94-8.80 (m, 4 H), 8.75 (s, 2 H), 8.10 (s, 2 H,), 7.80 - 7.72 (m, 4 H), 7.10 (s, 2 H), 7.01 (m, 2 H), 4.34 (s, 3 H), 4.07 (t, J = 6.6 Hz, 4 H), 4.03 (t, J = 6.3 Hz, 4 H), 2.87 (s, 3 H), 1.94 - 1.74 (m, 8 H), 1.60 - 1.40 (m, 8 H), 1.38-1.15 (m, 18 H), 1.12 (s, 42 H), 0.91 (t, J = 6.7 Hz, 6 H), 0.75 (t, J = 7.0 Hz, 6 H).

22a: According to the synthesis of **17** from **21a** (4.00 g; 3.80 mmol), THF (25 ml), H_2O (0.6 ml) and Bu_4NF (1 M in THF, 15.2 ml) **22a** (1.72 g, 60 %) was obtained after stirring for 48 h as an slightly orange solid that was used as received after one column chromatographic purification step (petroleum ether/CH_2Cl_2 (2:1), R_f = 0.61). ^{1}H-NMR (250 MHz, CD_2Cl_2): δ = 7.50 – 7.48 (m, 1 H), 7.32 – 7.30 (m, 2 H), 7.00 (s, 2 H), 6.99 (s, 2 H), 4.00 (t, J = 6.4 Hz, 4 H), 3.99 (t, J = 6.4 Hz, 4 H), 3.39 (s, 2 H), 2.36 (s, 3 H), 1.90-1.74 (m, 8 H), 1.62-1.27 (m, 24 H), 0.92 (t, J = 6.9 Hz, 6 H), 0.89 (t, J = 6.8 Hz, 6 H).

22b: According to the synthesis of **17** from **21b** (405 mg; 0.29 mmol), THF (5 ml) and Bu_4NF (1 M in THF, 1.8 ml) **22b** (313 mg, 99 %) was obtained after stirring for 20 h as an slightly yellow solid that was used as received after digestion with MeOH (two times) (petroleum ether/CH_2Cl_2 (2:1), R_f = 0.44). ^{1}H-NMR (250 MHz, CD_2Cl_2): δ = 9.02 (s, 2 H), 8.91 - 8.76 (m, 4 H), 8.71 (s, 2 H), 8.10 (s, 2 H,), 7.79 - 7.71 (m, 4 H), 7.13 (s, 2 H), 7.04 (s, 2 H), 4.34 (s, 3 H), 4.05 (t, J = 6.7 Hz, 4 H), 4.04 (t, J = 6.6 Hz,

4 H), 3.42 (s, 2 H), 2.86 (s, 3 H), 1.94-1.76 (m, 8 H), 1.60-1.18 (m, 24 H), 0.92 (t, *J* = 7.0 Hz, 6 H), 0.76 (t, *J* = 7.1 Hz, 6 H).

23a: According to the general procedure for the preparation of the diiodo compounds **6** from **22a** (501 mg, 0.68 mmol), **5c** (3.30 g, 5.39 mmol), $Pd(PPh_3)_2Cl_2$ (14 mg), CuI (7 mg) and PPh_3 (14 mg) in THF/piperidine (5/1, 12 ml) **21a** was obtained after stirring for 16 h at 60 °C as a slightly yellow solid (556 mg, 48 %) (petroleum ether/CH_2Cl_2 (4:1), R_f = 0.40).^{1}H-NMR (250 MHz, $C_2D_2Cl_4$): δ = 7.61 – 7.58 (m, 2 H), 7.56 – 7.53 (m, 1 H), 7.36 – 7.34 (m, 2 H), 7.31 – 7.28 (m, 2 H), 7.01 (s, 2 H), 6.99 (s, 2 H), 4.17 (t, *J* = 6.7 Hz, 4 H), 4.03 (t, *J* = 6.4 Hz, 4 H), 2.37 (s, 3 H), 2.27 (s, 6 H), 1.93-1.77 (m, 12 H), 1.64-1.20 (m, 84 H), 0.90 (t, *J* = 7.1 Hz, 12 H), 0.88 (t, *J* = 7.2 Hz, 6 H). MS (FAB): 1708.9 [M+].

23b: According to the general procedure for the preparation of the diiodo compounds **6** from **22b** (209 mg, 0.19 mmol), **5c** (1.09 g, 1.83 mmol), $Pd(PPh_3)_2Cl_2$ (10 mg), CuI (6 mg) and PPh_3 (11 mg) in THF/piperidine (2/1, 11 ml) **21a** was obtained after 48 h stirring at room temperature as a slightly yellow oil (183 mg, 46 %) (petroleum ether/CH_2Cl_2 (2:1), R_f = 0.38). ^{1}H-NMR (250 MHz, CD_2Cl_2): δ = 9.13 (s, 2 H), 8.98 - 8.84 (m, 4 H), 8.80 (s, 2 H), 8.13 (s, 2 H,), 7.82 - 7.76 (m, 4 H), 7.63 - 7.60 (m, 2 H), 7.32 – 7.29 (m, 2 H), 7.16 (s, 2 H), 7.06 (s, 2 H), 4.36 (s, 3 H), 4.18 (t, *J* = 6.5 Hz, 4 H), 4.08 (t, *J* = 6.5 Hz, 8 H), 2.90 (s, 3 H), 2.28 (s, 6 H), 1.96 - 1.78 (m, 12 H), 1.62 - 1.14 (m, 84 H), 0.89 (t, *J* = 6.9 Hz, 6 H), 0.84 (t, *J* = 6.9 Hz, 6 H), 0.76 (t, *J* = 7.1 Hz, 6 H).

24a: According to the general procedure for the preparation of **7** from **23a** (380 mg, 0.22 mmol), TMS acetylene (0.21 g, 2.16 mmol), $Pd(PPh_3)_2Cl_2$ (5 mg), CuI (2.5 mg) and PPh_3 (5 mg) in piperidine (12 ml) **24a** was obtained after stirring for 16 h at 60 °C as a slightly yellow solid (361 mg, 98 %) (petroleum ether/CH_2Cl_2 (2:1), R_f = 0.61). ^{1}H-NMR (250 MHz, $C_2D_2Cl_4$): δ = 7.51 – 7.48 (m, 1 H), 7.35 – 7.32 (m, 2 H), 7.28 – 7.26 (m, 2 H), 7.24 – 7.22 (m, 2 H), 7.03 (s, 2 H), 7.01 (s, 2 H), 4.24 (t, *J* = 6.5 Hz, 4 H), 4.04 (t, *J* = 6.5 Hz, 8 H), 2.37 (s, 3 H), 2.27 (s, 6 H), 1.92 - 1.74 (m, 12 H), 1.65 - 1.15 (m, 84 H), 0.90 (t, *J* = 9.9 Hz, 12 H), 0.87 (t, *J* = 6.9 Hz, 6 H), 0.27 (s, 18 H). MS (FAB): 1649.7 [M+].

24b: According to the general procedure for the preparation of **7** from **23b** (175 mg, 0.09 mmol), TIPS acetylene (0.10 g, 0.52 mmol), $Pd(PPh_3)_2Cl_2$ (6 mg), CuI (4 mg) and PPh_3 (6 mg) in THF/piperidine (2/1, 6 ml). **24a** was obtained after stirring for 24 h at room temperature as a yellow oil (114 mg, 64 %) (petroleum ether/CH_2Cl_2 (2:1), R_f = 0.35). ^{1}H-NMR (250 MHz, CD_2Cl_2): δ = 9.15 (s, 2 H), 8.98 - 8.84 (m, 4 H), 8.82 (s, 2 H), 8.14 (s, 2 H,), 7.84 - 7.77 (m, 4 H), 7.30 – 7.24 (m, 4 H), 7.15 (s, 2 H), 7.06 (s, 2 H), 4.35 (s, 3 H), 4.26 (t, *J* = 6.8 Hz, 4 H), 4.12 – 4.04 (m, 8 H), 2.91 (s, 3 H), 2.28 (s, 6 H), 1.93 - 1.78 (m, 12 H), 1.65 - 1.10 (m, 84 H), 1.16 (s, 42 H), 0.90 - 0.78 (m, 12 H), 0.75 (t, *J* = 7.0 Hz, 6 H).

25a: According to the general procedure for the preparation of **8** from **24a** (330 mg, 0.20 mmol), K_2CO_3 (87 mg, 0.63 mmol), THF (10 ml) and MeOH (10 ml) **25a** was obtained after stirring for 16 h as a yellow oil (286 mg, 95 %) (petroleum ether/CH_2Cl_2 (2:1), R_f = 0.22). ^{1}H-NMR (250 MHz, $C_2D_2Cl_4$): δ = 7.51 – 7.48 (m, 1 H), 7.35 – 7.33 (m, 2 H), 7.32 – 7.30 (m, 2 H), 7.27 – 7.24 (m, 2 H), 7.03 (s, 2 H), 7.02 (s, 2 H), 4.25 (t, *J* = 6.4 Hz, 4 H), 4.04 (t, *J* = 6.5 Hz, 8 H), 3.30 (s, 2 H), 2.38 (s, 3 H), 2.28 (s, 6 H), 1.92 - 1.77 (m, 12 H), 1.64 - 1.18 (m, 84 H), 0.90 (t, *J* = 6.9 Hz, 12 H), 0.87 (t, *J* = 6.8 Hz, 6 H). MS (FAB): 1505.5 [M+]. GPC: single peak at M_w = 1980 g mol^{-1}.

25b: According to the synthesis of **17** from **24b** (110 mg; 0.05 mmol), THF (2 ml) and Bu_4NF (1 M in THF, 0.4 ml) **25b** (94 mg, 99 %) was obtained after stirring for 2.5 h at room temperature as a yellow solid that was used as received after digestion with MeOH (two times) (petroleum ether/CH_2Cl_2 (2:1), R_f = 0.47). ^{1}H-NMR (250 MHz, CD_2Cl_2): δ = 9.14 (s, 2 H), 9.00 - 8.82 (m, 4 H), 8.80 (s, 2 H), 8.13 (s, 2 H,), 7.82 - 7.74 (m, 4 H), 7.34 – 7.31 (m, 2 H), 7.28 – 7.25 (m, 2 H), 7.16 (s, 2 H), 7.07 (s, 2 H), 4.36 (s, 3 H), 4.26 (t, *J* = 6.5 Hz, 4 H), 4.09 (t, *J* = 6.6 Hz, 8 H), 3.31 (s, 2 H), 2.90 (s, 3 H), 2.29 (s, 6 H), 1.98-1.73 (m, 12 H), 1.62-1.05 (m, 84 H) 0.90 (t, *J* = 7.0 Hz, 6 H), 0.84 (t, *J* = 7.0 Hz, 6 H), 0.76 (t, *J* = 7.1 Hz, 6 H). GPC: single peak at M_w = 2050 g mol^{-1}.

26a: According to the general procedure for the preparation of **8** from **25a** (270 mg, 0.18 mmol) in pyridine (25 ml) and CuCl (0.98 g) and $CuCl_2$ (0.20 g) in pyridine (25 ml). **26a** (62 mg, 23 %) (petroleum ether/CH_2Cl_2 (2:1), R_f = 0.12) was obtained after purification by preparative GPC. ^{1}H-NMR (250 MHz, $C_2D_2Cl_4$): δ = 7.55 – 7.52 (m, 2

H), 7.34 – 7.31 (m, 8 H), 7.29 – 7.27 (m, 4 H), 7.04 (s, 4 H), 7.03 (s, 4 H), 4.31 (t, *J* = 6.4 Hz, 8 H), 4.04 (t, *J* = 6.5 Hz, 16 H), 2.38 (s, 6 H), 2.30 (s, 12 H), 1.94 - 1.88 (m, 24 H), 1.65 - 1.14 (m, 168 H), 0.96 - 0.80 (m, 36 H). MS (MALDI-TOF): 3010.2 [M+]. GPC: single peak at M_w = 2750 g mol^{-1}.

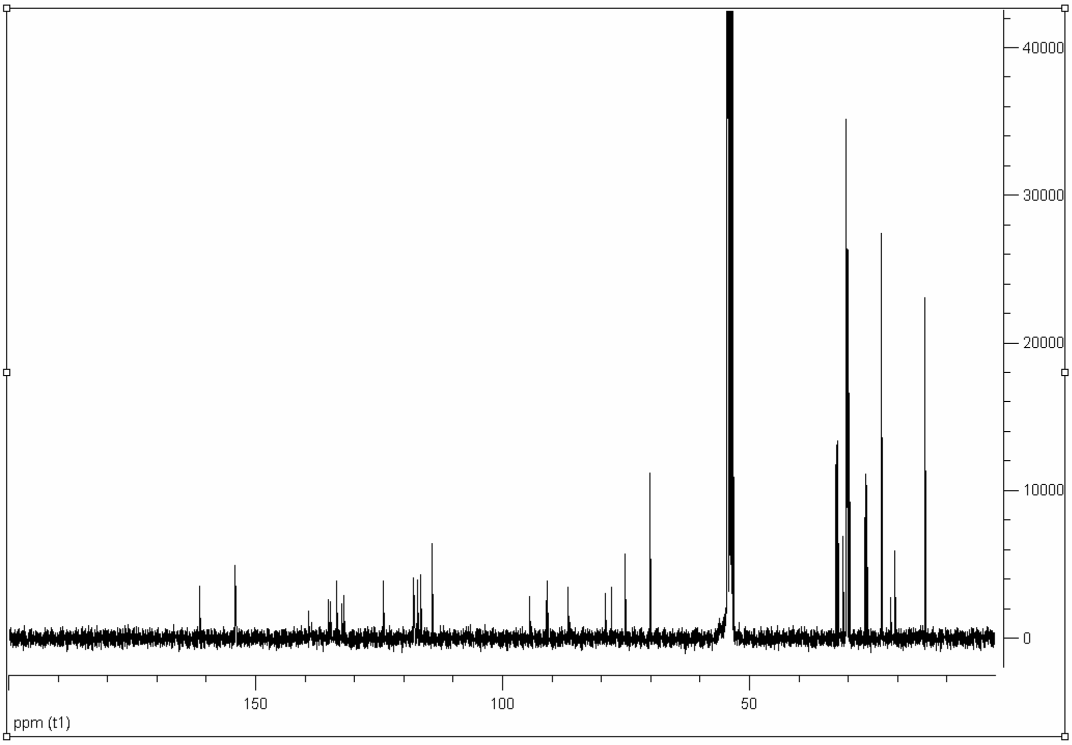

13C NMR spectrum of **26a.**

26b: According to the general procedure for the preparation of **8** from **25b** (97 mg, 0.05 mmol) in pyridine (20 ml) and CuCl (0.30 g) and $CuCl_2$ (0.04 g) in pyridine (40 ml). **26b** (6 mg, 7 %) (petroleum ether/CH_2Cl_2 (1:1), R_f = 0.37) was obtained after purification by preparative GPC. ^{1}H-NMR (250 MHz, CD_2Cl_2): δ = 9.11 (s, 4 H), 8.98 - 8.82 (m, 8 H), 8.78 (s, 4 H), 8.13 (s, 4 H), 7.83 - 7.73 (m, 8 H), 7.36 - 7.27 (m, 8 H), 7.18 (s, 4 H), 7.07 (s, 4 H), 4.36 (s, 6 H), 4.34 (t, *J* = 6.4 Hz, 8 H), 4.11 (t, *J* = 6.7 Hz, 8 H), 4.10 (t, *J* = 6.5 Hz, 8 H), 2.89 (s, 6 H), 2.32 (s, 12 H), 1.98-1.82 (m, 24 H), 1.68-1.10 (m, 168 H) 0.92 (t, *J* = 7.0 Hz, 12 H), 0.84 (t, *J* = 6.9 Hz, 12 H), 0.81 (t, *J* = 6.9 Hz, 12 H). MS (FAB): 3668.2 [M+]. GPC: single peak at M_w = 2950 g mol^{-1}. Due to the limited amount of material we were not able to obtain a useful 13C NMR spectrum.

As with other compounds containing PAH units, an aggregation of the molecules in solution could be observed. By assuming a monomer-dimer equilibrium as dominant

process in solution an aggregation constant K of 13 M^{-1} could be extracted from the concentration dependent ^{1}H-NMR data. Since a direct overlap of the macrocycles seems to be sterically unfavorable, higher aggregation constants may lead to a staircase-like arrangement of the molecules.

References

[1] Wheeler H L, Liddle L M: *Am. Chem. J.* 1910, **42**: 441-461.
[2] Ref 29 of the manuscript.

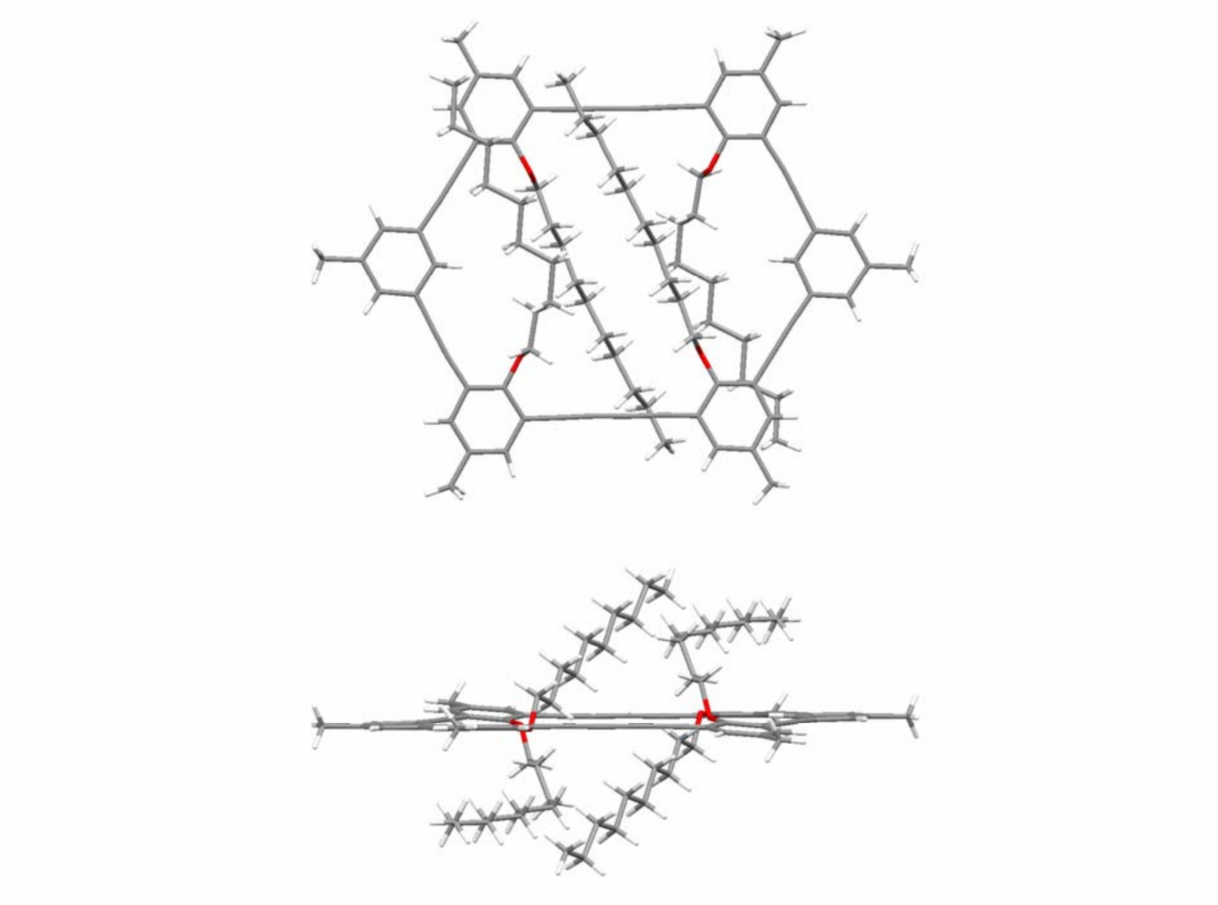

Top and side view of one of the crystallographic independent macrocycles.

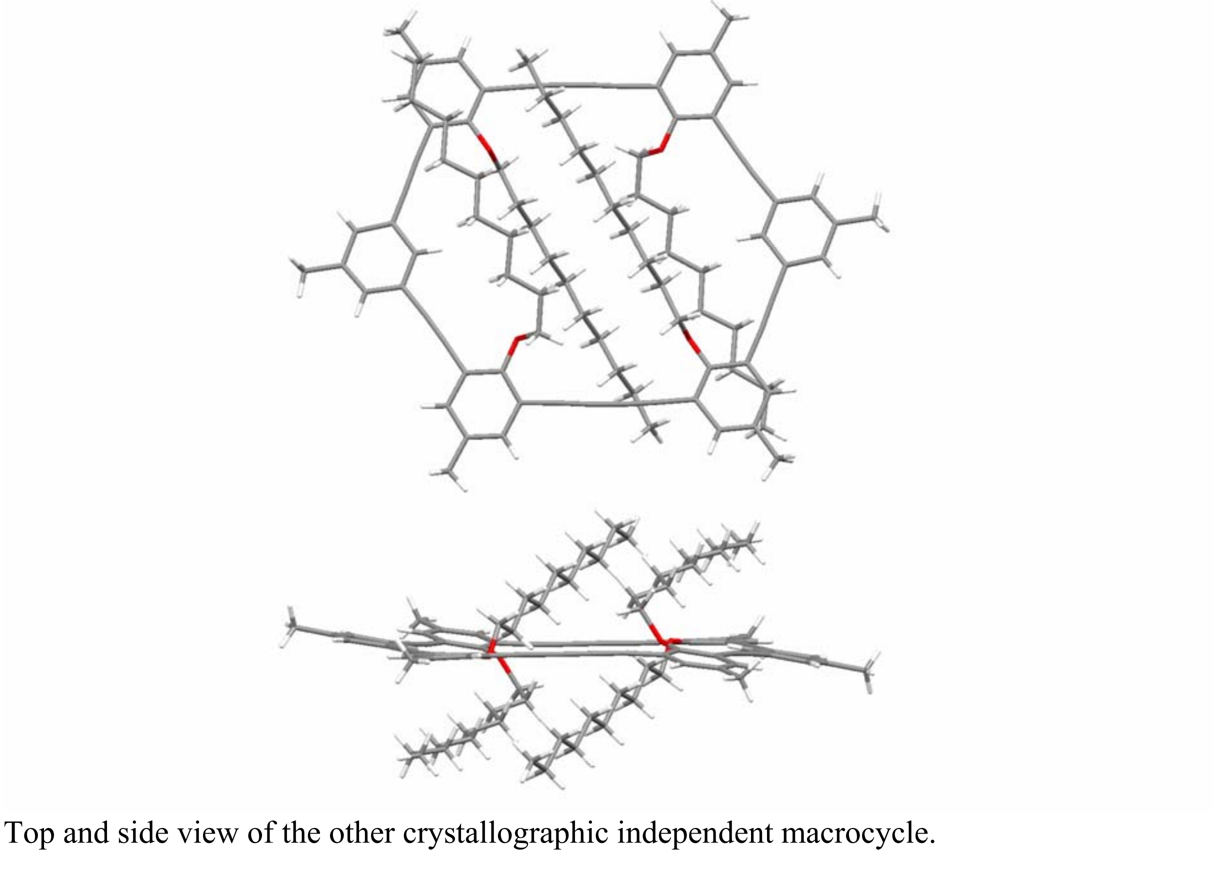

Top and side view of the other crystallographic independent macrocycle.

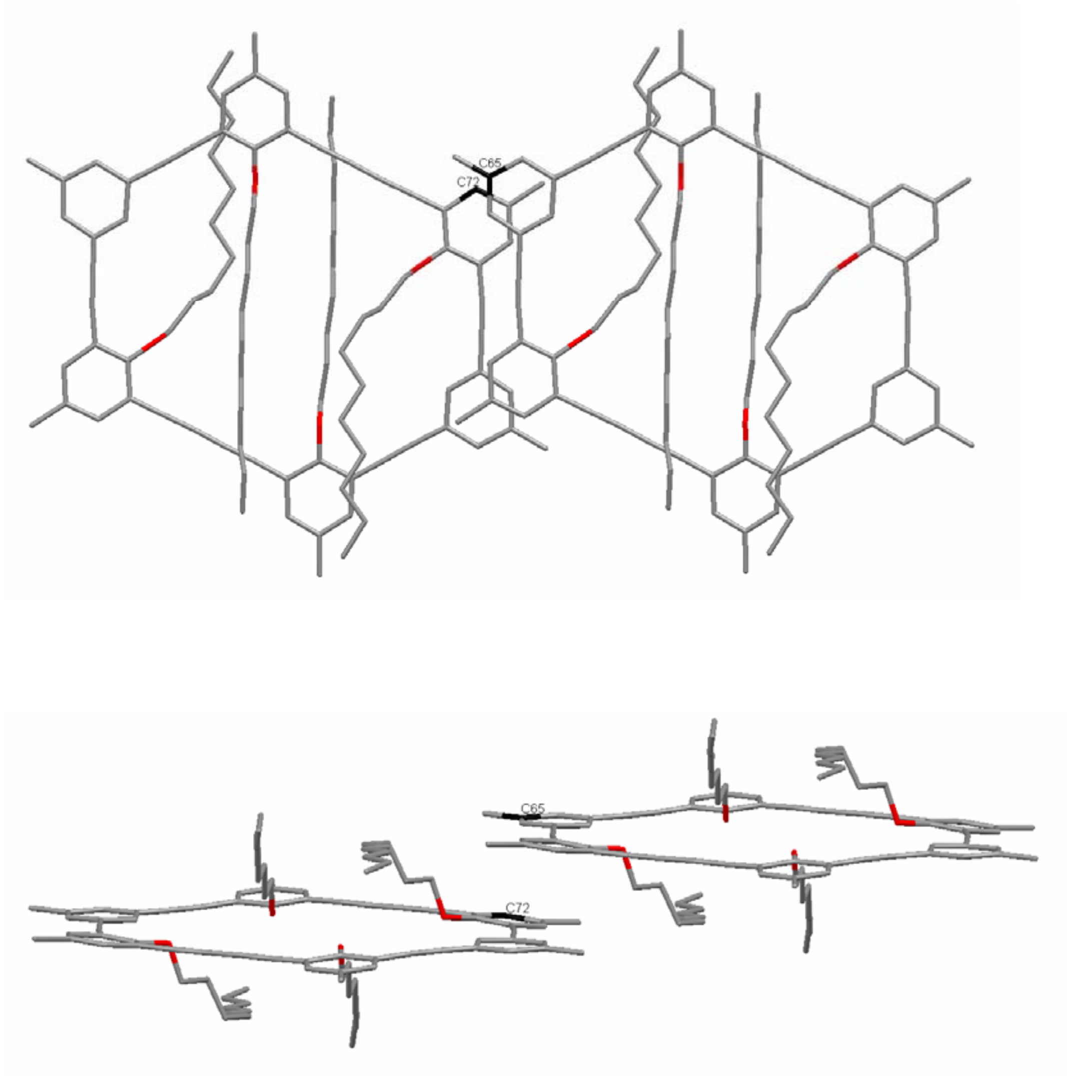

Top and side view of two macrocycles with close contacts in the solid state (including relevant atoms labelled in black; H atoms are not displayed).
